# Conditions that promote transcellular neutrophil migration in vivo

**DOI:** 10.1038/s41598-024-65173-3

**Published:** 2024-06-24

**Authors:** Min Xia, Rebekka I. Stegmeyer, Keisuke Shirakura, Stefan Butz, Aude Thiriot, Ulrich H. von Andrian, Dietmar Vestweber

**Affiliations:** 1 Department of Vascular Cell Biology, Max Planck Institute for Molecular Biomedicine, Röntgenstr. 20, 48149 Münster, Germany; 2 Department of Immunology and Center for Immune Imaging, Harvard Medical School, Boston, MA USA

**Keywords:** Leukocyte extravasation, Endothelium, Cell adhesion, Inflammation, Imaging the immune system, Cell biology, Immunology

## Abstract

Circulating leukocytes enter tissue either through endothelial junctions (paracellular) or via a pore through the body of endothelial cells (transcellular). We have previously shown that genetically replacing VE-cadherin with a VE-cadherin-α-catenin (VEC-αC) fusion construct—which binds constitutively to actin—obstructs junctions, and blocks leukocyte extravasation in lung, skin and postcapillary venules of cremaster muscle. However, neutrophil recruitment into the inflamed peritoneal cavity was unimpaired. Investigating reasons for this, here, we visualized neutrophil diapedesis by 3D intravital video microscopy in the cremaster muscle and omentum, the major site of neutrophil recruitment into the peritoneal cavity. We found that 80% of neutrophil-extravasation occurred through HEVs in the omentum, which was unimpaired by VEC-αC. In addition, in larger venules (60–85 µm) of both tissues, less than 15% of neutrophils extravasated transcellularly in WT mice. However, in VEC-α-C mice, transcellular diapedesis increased severalfold in the omentum, but not in the cremaster. In line with this, omental venules expressed higher levels of ICAM-1 and atypical chemokine receptor 1. Furthermore, only in the omentum, VEC-αC expression caused reduced elongation of venular endothelium in flow-direction, suggesting different biomechanical properties. Collectively, VEC-αC does not inhibit paracellular transmigration in all types of venules and can modulate the diapedesis route.

## Introduction

Endothelial cells represent the crucial barrier for the entry of leukocytes into tissue, a central step to clear infections and initiate tissue repair. Leukocyte recruitment is initiated by capturing to the luminal surface of microvascular endothelium, which is mediated by an interplay of adhesion molecules and chemokines. This is followed by the actual diapedesis process, the migration through the endothelial barrier. Diapedesis occurs via two different routes: through the junctions between endothelial cells (paracellular route) and directly via a pore through the body of endothelial cells (transcellular route)^1,2^.

In vitro studies have documented that transendothelial migration of leukocytes occurs preferentially through the paracellular route (70–90%) and only at low frequency through the transcellular route^1–4^. In vivo this was most convincingly demonstrated by 3D intravital video microscopy of the mouse cremaster muscle where 90% of extravasating neutrophils used the paracellular diapedesis route independent of the type of inflammatory stimulus employed^5^.

The endothelium of the blood brain barrier was initially thought to represent a special case due to elaborated tight junctions. However, T cells seem to cross the BBB at least under low inflammatory conditions preferentially via the paracellular route^6,7^ and only more severe inflammatory conditions seem to increase the efficiency of the transcellular migration mechanism.

It is not well understood what determines the type of diapedesis route through which leukocytes transmigrate. For endothelium of the blood brain barrier, it was reported that an increase in the expression of the atypical chemokine receptor 1 (ACKR1) favored the transcellular over paracellular migration of T cells across cultured endothelial cells^8^. Likewise, overexpression of ICAM-1 in cultured EC leads to increased transcellular migration efficiency^9^. In agreement with this, the expression level of ICAM-1 on brain endothelial cells correlated with the efficiency of transcellular T cell diapedesis in vitro^6^. It was suggested that the interaction of leukocytes with endothelial ICAM-1 triggers the clustering of this adhesion molecule which results in the recruitment of vesiculo-vacuolar organelles (VVO) that form a channel through the cell which serves as a pore for transmigration^10,11^. In addition, vimentin and the membrane protein PV-1 were also shown to support transcellular migration^12,13^.

Quantification of paracellular and transcellular migration of leukocytes in vivo is still technically demanding and requires visualization of endothelial junctions during leukocyte transmigration. This was best analyzed by 3D live intravital confocal microscopy in the cremaster muscle^5^. Since this sophisticated technique is not generally applicable for all organs with similar quality, we have previously established a genetic approach to investigate the relevance of the paracellular migration route for a broader range of organs. For this, we established knock-in mice expressing a modified version of VE-cadherin, which was fused to α-catenin and highly stabilized endothelial junctions^14^. VE-cadherin is a major adhesion molecule of endothelial junctions, which controls neutrophil extravasation^15–17^. Linkage of VE-cadherin via β- and α-catenin to the actin cytoskeleton is required for the integrity of endothelial junctions. A fusion construct made of VE-cadherin and α-catenin (VEC-αC) binds more avidly to actin due to conformational changes in α-catenin^18^ and blocks paracellular diapedesis of neutrophils through HUVEC monolayers^14^. Replacing VE-cadherin by VEC-αC in knock in mice made these mice resistant to the induction of vascular permeability in the skin. In addition, neutrophil or lymphocyte recruitment into inflamed cremaster, lung and skin were strongly inhibited^14^. This was a compelling argument for the paracellular pathway as main exit route for extravasating leukocytes in these tissues.

However, homing of naïve lymphocytes into lymph nodes (which occurs through high endothelial venules (HEVs)) and neutrophil recruitment into the IL-1β stimulated peritoneal cavity was not inhibited in VEC-αC knock-in mice^14,19^. The reason for this is unexplained.

Here, we have investigated why replacing VE-cadherin by VEC-αC in gene targeted mice does not interfere with neutrophil recruitment into the inflamed peritoneal cavity whereas neutrophil recruitment in postcapillary venules of the cremaster and into other tissues was strongly blocked. To understand the differences between cremaster and peritoneum, we performed 3D live intravital microscopy and quantified paracellular and transcellular diapedesis of neutrophils. We found that in the greater omentum, the main entry site of neutrophils into the inflamed peritoneal cavity^20,21^, neutrophil extravasation occurred mainly through HEVs in milky spots and was not inhibited by VEC-αC. In larger venules (60–85 µm) where trans- and paracellular diapedesis could be optically distinguished, we found that the junctional exit pathway was the major route in WT mice, similar to the cremaster. However, in VEC-αC mice, the efficiency of transcellular extravasation of neutrophils was increased severalfold in venules of the omentum, but not in the cremaster. Additional characteristics such as elevated expression levels of ICAM-1 and ACKR1 and different mechanobiological properties of omental venules may contribute to their different functioning in leukocyte extravasation in comparison to venules in the cremaster.

## Results

### Increasing the interaction between VE-cadherin and junctional actin boosts transcellular diapedesis of neutrophils in omentum, but not in cremaster muscle

**Figure 1:**
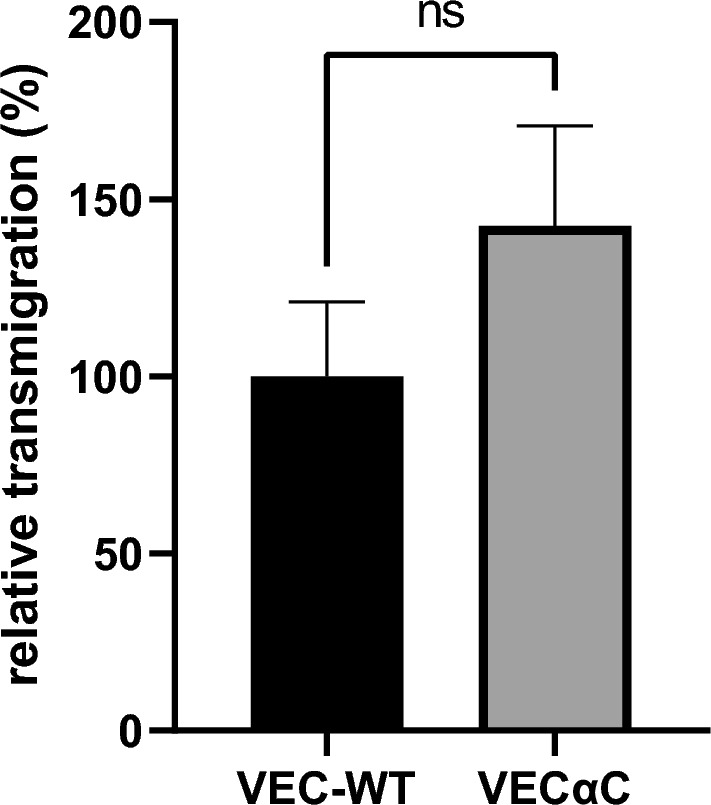
VEC-αC does not impair neutrophil recruitment into inflamed peritoneal cavity. Number of neutrophils isolated from the peritoneal lavage 3 h after i.p. injection of 50 ng IL-1β in VEC-WT or VEC-αC mice. Data were analyzed from 4 different experiments, with 16 to 17 mice in total and are presented as mean ± SEM. ns P = 0.3824; t-test.

We have shown previously that knock-in mice expressing a VE-cadherin-α-catenin (VEC-αC) fusion protein instead of VE-cadherin have highly stabilized endothelial junctions which resist destabilization in multiple inflammatory settings and tissues^14^. Consequently, VEGF and histamine induced vascular permeability in the skin was completely blocked in these mice and neutrophil recruitment into the IL-1β stimulated cremaster (3 h) and LPS challenged lung (4 h) were inhibited by 74% and 63%, respectively^14^. In contrast, neutrophil recruitment into the IL-1ß stimulated peritoneal cavity (2 h) of VEC-αC mice was not reduced in comparison to WT mice^19^. To determine, why VEC-αC did not block neutrophil recruitment in the peritonitis model, we first repeated these experiments, this time after 3 h i.p. stimulation with 50 ng IL-1β. Again, we found no significant difference in the number of neutrophils recruited to the peritoneal cavity in WT and VEC-αC mice (Fig. 1).

To understand the reason for this tissue specific lack of inhibition, we decided to investigate the diapedesis process directly by intravital video microscopy and determine the number of extravasating neutrophils that exit through the transcellular and the paracellular route in cremaster and omentum in WT and VEC-αC mice. We chose the omentum since this has been reported to be the major site of neutrophil recruitment into the inflamed peritoneal cavity^20,21^.

**Figure 2:**
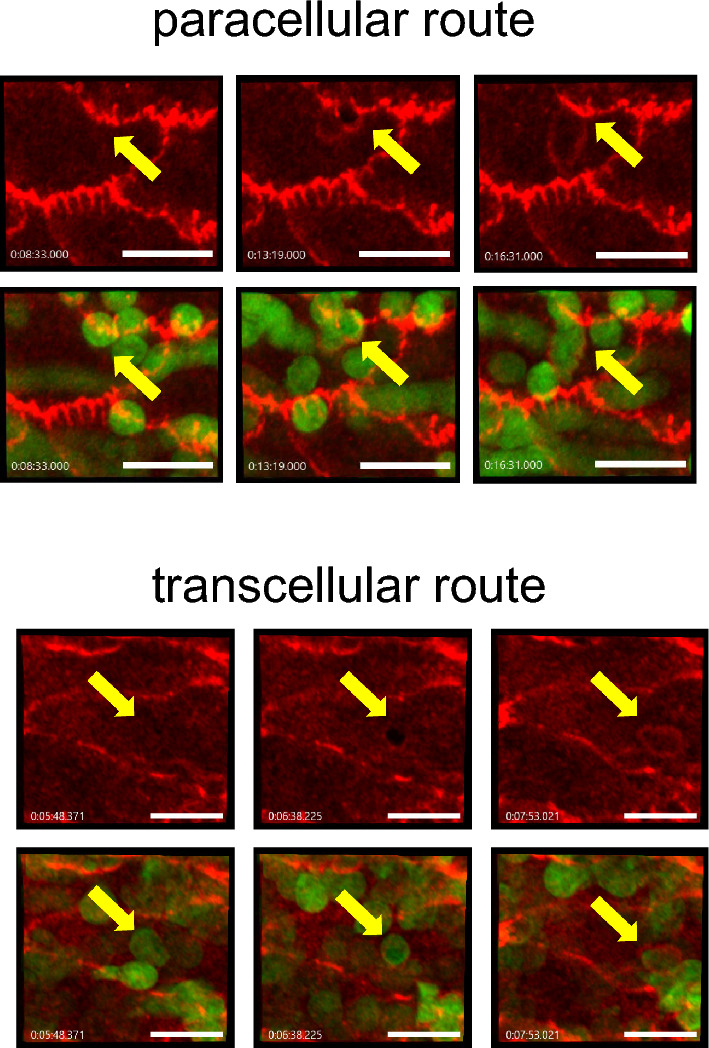
Examples for paracellular and transcellular diapedesis events. Images from different time points (min) of videos documenting paracellular and transcellular neutrophil diapedesis events in venules of 60–85 µm diameter. Endothelial junctions are stained in red for PECAM-1, neutrophils are genetically labeled by LysM-EGFP (green). Arrows point to sites of neutrophil transmigration. Bar = 20 µm.

To distinguish paracellular from transcellular extravasation events, we labeled endothelial junctions by i.v. injection of a directly labeled anti-PECAM-1 mAb and observed extravasation by 3D intravital confocal laser scanning microscopy in real time of the cremaster and the omentum. Leukocytes were genetically labeled by EGFP. The first wave of neutrophil extravasation in the omentum occurs through high endothelial venules (HEV) of so called “milky spots”, lymphoid follicle-like sites in the omentum (supplemental video 1)^21^. Due to their structure and the massive stream of exiting neutrophils, it was not possible to clearly distinguish paracellular from transcellular migration routes in this special type of vessels. Therefore, we analyzed larger venules with 60–85 µm diameter, where the number of extravasating neutrophils is initially smaller than in HEV, before the numbers catch up at later time points. For comparison, we analyzed venules of similar size in the cremaster. Figure 2 shows typical examples for paracellular and transcellular extravasation events, which were picked from supplemental videos 4–7. Neutrophils that used the paracellular pathway caused reversible opening of junctions (supplemental videos 4 and 5), whereas neutrophils exiting through the transcellular route left junctions intact (supplemental videos 6 and 7). Supplemental videos 4–7 zoom into smaller areas, which were chosen from larger overviews that are shown in supplemental videos 2 and 3.

**Figure 3:**
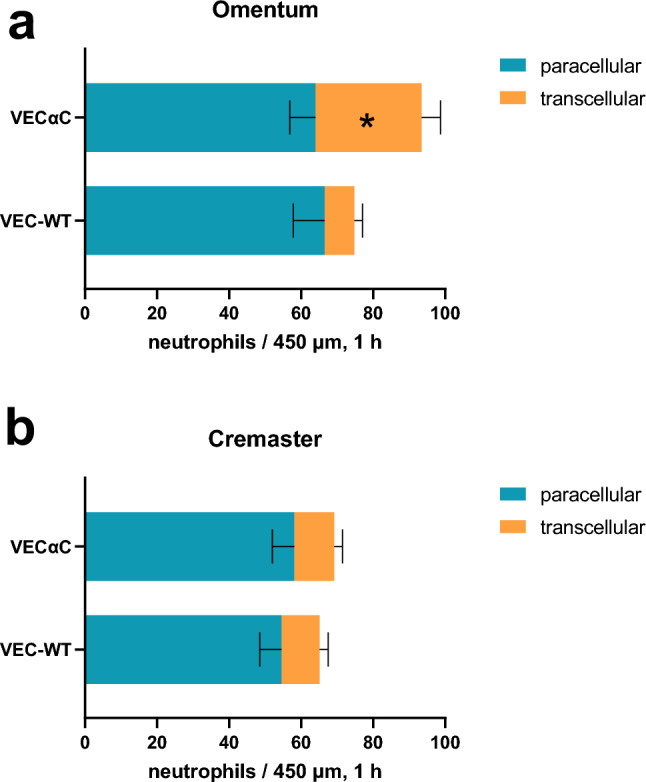
Quantification of paracellular and transcellular diapedesis of neutrophils in vivo. Number of paracellular (blue) and transcellular (red) neutrophil diapedesis events (per 450 µm and per hour) in venules of the omentum (**a**) and the cremaster muscle (**b**) of VEC-αC mice and VEC-WT mice (as indicated on the left). A total of 88 vessel segments (diameter range from 60 to 85 μm), 1289 events, were evaluated. (n = 26 VEC-WT cremaster, n = 23 VEC-αC cremaster, n = 18 VEC-WT omentum, n = 21 VEC-αC omentum). *P = 0.029; two-way ANOVA.

For quantification, we collected results from at least 18 vessel segments from the omentum as well as from the cremaster muscle with vessel diameters ranging between 60 and 85 µm. Based on this analysis, we found that in WT mice, the paracellular transmigration route was the dominant diapedesis pathway in the cremaster as well as in the omentum. In the cremaster, this pathway was used by 84% of extravasating neutrophils and in the omentum by 89% (calculated from Fig. 3). Interestingly, transcellular migration of neutrophils in the omentum increased from 11% in WT mice to 31.5% in VEC-αC mice (calculated from Fig. 3a). In contrast, in the cremaster the transcellular route was used by 16% of neutrophils in WT mice and by 15% in VEC-αC mice (Fig. 3b). Thus, increasing the efficiency of the interaction of VE-cadherin with junctional actin leads to an almost threefold increase of transcellular migration in the omentum, whereas the transcellular diapedesis is unaffected by actin anchorage of VE-cadherin in cremaster venules. It is likely, that the increase in transcellular migration events in the omentum of VEC-αC mice contributes to the lack of inhibition of neutrophil recruitment into the inflamed peritoneal cavity in these mice.

A second surprising finding that is revealed by our results was that VEC-αC did not inhibit paracellular extravasation in larger venules (60–85 µm diameter) of the cremaster. This is in contrast to our results in cremaster venules of smaller caliber (20–35 µm)^14^. Thus, the molecular mechanisms which control the paracellular pathway in the cremaster seem to differ between postcapillary and slightly larger venules.

### Does VEC-αC inhibit PMN extravasation through HEV-like vessels in omental milky spots?

**Figure 4:**
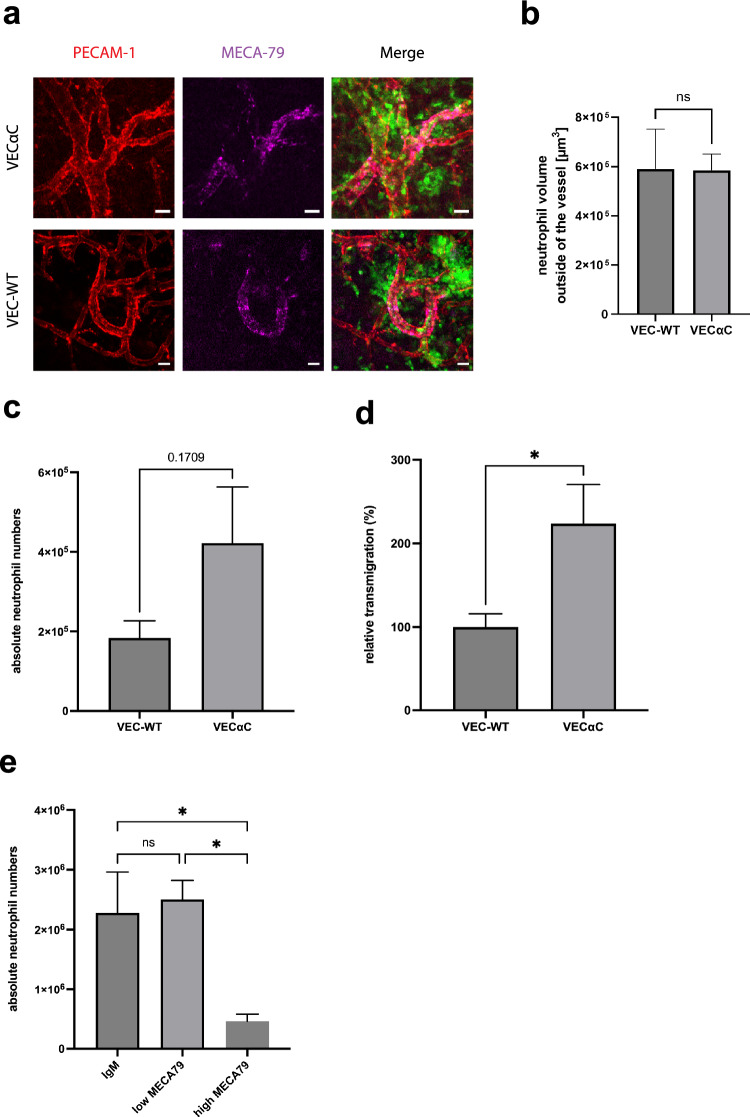
VEC-αC does not inhibit PMN extravasation through omental HEVs, but increases extravasation through larger, non-HEV venules. (**a**) Confocal microscopic images of HEVs in the omentum of VEC-WT and VEC-αC mice. Endothelial junctions are stained for PECAM-1 (red), high endothelial venules (HEV) in milky spots by MECA79 (magenta) and neutrophils are genetically labeled by LysM-EGFP (green). Bar = 20 µm. (**b**) Quantification of the volume occupied by neutrophils outside of HEVs 3 h after IL-1β stimulation in VEC-WT or VEc-αC mice. A total of 29 vessels were evaluated (n = 5 mice VEC-WT, n = 4 mice VEc-αC). P = 0.9717, t-test. (**c** and **d**) Absolute numbers (**c**) and relative numbers (**d**) of neutrophils isolated from the peritoneal lavage of VEC-WT or VEC-αC mice treated with 50 µg anti-MECA79 antibody to inhibit leukocyte extravasation through HEVs. 3 h after i.p. injection of 50 ng IL-1β peritoneal exudates were collected. Data were analyzed from 3 different experiments, with 9–12 mice in total and are presented as mean ± SEM. * P = 0.0396; t-test. (**c** and **d**) are based on the same set of results. (**e**) Number of neutrophils isolated from the peritoneal lavage of C57Bl/6 mice treated with 3 µg (non-blocking dose, used for staining) or 50 µg anti-MECA79 antibody (blocking dose, inhibition of leukocyte extravasation through HEVs). 3 h after i.p. injection of 50 ng IL-1 peritoneal exudates were collected. Data were analyzed from 9 to 10 mice per group (28 mice in total) and are presented as mean ± SEM. * P ≤ 0.05; one-way ANOVA.

Since the increase in transcellular migration, that we found in larger omental venules was limited, it may contribute but not fully explain the lack of inhibition of neutrophil accumulation in the peritoneal cavity of VEC-αC mice. Therefore, we tested the alternative explanation that VEC-αC might fail to block extravasation through HEV-like vessels in milky spots, the sites of the first wave of exiting leukocytes. To this end, we compared the extravasation of neutrophils from HEV-like vessels in the omentum of WT and VEC-αC mice by intravital microscopy. As shown in Fig. 4a and b, we found that the overall extravasation within 3 h after IL-1β stimulation was largely similar for both genotypes. To determine the relevance of HEV-like vessels for the overall recruitment of PMNs into the inflamed peritoneal cavity, we repeated peritonitis experiments in WT and mutant mice (similar as for Fig. 1), except this time we blocked the extravasation through HEV-like vessels with the anti-vascular addressin mAb MECA79. Under these conditions, we found again an increase of peritoneal neutrophils accumulating in VEC-αC mice when compared with WT mice (Fig. 4c and d), in line with the increase in transcellular extravasation efficiency in larger, non HEV venules (Fig. 3). However, this increase was small compared to the loss of peritoneal neutrophil accumulation caused by blocking the HEV pathway (compare numbers of peritoneal neutrophils recruited in mice treated with or without MECA79 (Fig. 4e)). In other words, the major reason why there is no significant block of neutrophil accumulation in the peritoneal cavity of VEC-αC mice is probably due to a lack of inhibition of HEV-mediated extravasation in the omentum.

### Venules in omentum express higher levels of ICAM-1 and ACKR1 than in cremaster

**Figure 5:**
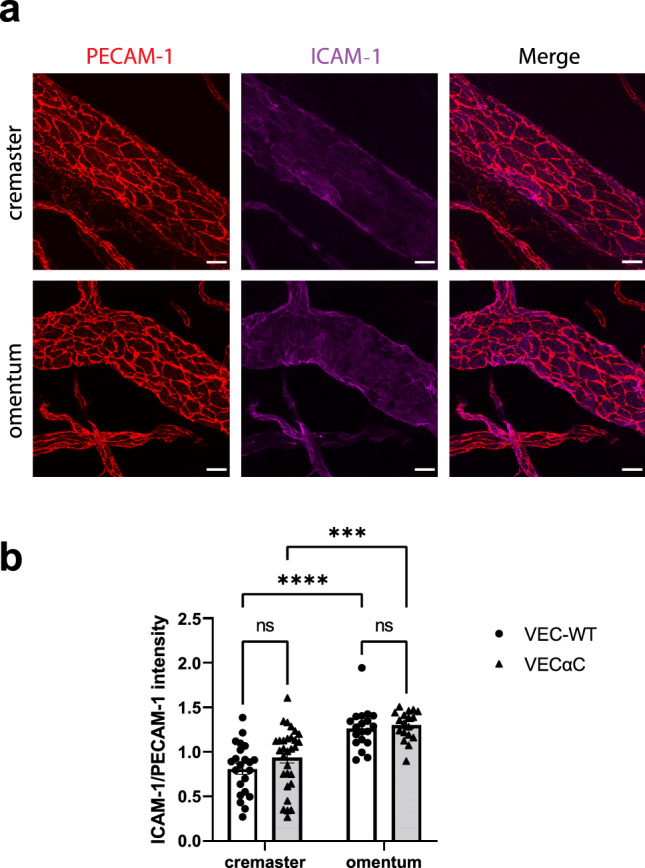
Expression levels of ICAM-1 on venular endothelium of omentum and cremaster. (**a**) Confocal microscopic images of venules in the cremaster muscle or the omentum (as indicated on the left) stained for PECAM-1 (red) and ICAM-1 (magenta); bar = 20 µm. (**b**) Quantification of staining intensity for ICAM-1 normalized for the staining intensity of PECAM-1 of a total of 89 vessel segments (n = 22 VEC-WT cremaster, n = 30 VEC-αC cremaster, n = 19 VEC-WT omentum, n = 18 VEC-αC omentum), presented as mean ± SEM. All vessel segments had diameters of 60–85 µm. ***P < 0.0008, ****P < 0.0001; two-way ANOVA.

Based on in vitro studies, it was shown that higher expression levels of ICAM-1 correlated with increased transcellular migration of neutrophils^9^ and T cells^8^. Therefore, we compared the expression levels of ICAM-1 on venules of cremaster and omentum of WT and VEC-αC mice by indirect immunofluorescence staining of tissue whole mounts. Figure 5a illustrates the staining for ICAM-1 and PECAM-1 of cremaster and omentum venules. Quantification of staining intensities over a total of 89 venules of both tissues (after IL-1β stimulation) and both genotypes revealed a significant increase of the expression of ICAM-1 in the omentum, compared to cremaster muscle, whereas the genotype (WT versus VEC-αC) made no difference (Fig. 5b).

**Figure 6:**
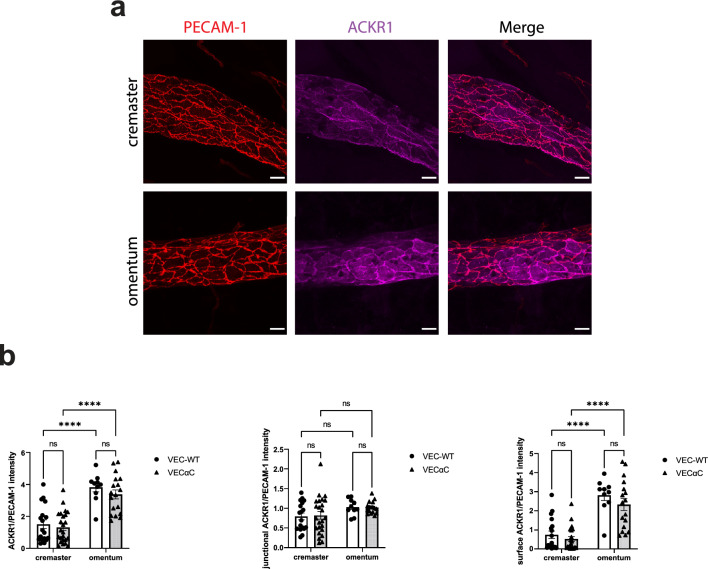
Expression levels of ACKR1 on venular endothelium of omentum and cremaster. Confocal microscopic images of venules in the cremaster muscle or the omentum (as indicated on the left) stained for PECAM-1 (red) and ACKR1 (magenta); bar = 20 µm. (**b**) Quantification of staining intensity for ACKR1 normalized for the staining intensity of PECAM-1 was determined for total ACKR1 (left graph), for junctional ACKR1 (middle graph) and for surface ACKR1 outside of junction areas (right graph). A total of 70 vessel segments were evaluated (n = 18 VEC-WT cremaster, n = 24 VEC-αC cremaster, n = 10 VEC-WT omentum, n = 18 VEC-αC omentum) and presented as mean ± SEM. All vessel segments had diameter ranges from 60 to 85 μm. ****P < 0.0001; two-way ANOVA.

A similar analysis was performed for the atypical chemokine receptor 1 (ACKR1), for which high expression has been shown to correlate with transcellular migration of lymphocytes. Again, we found that ACKR1 expression was higher in venules of the omentum compared to venules in the cremaster, whereas no differences were found between WT and VEC-αC mice (Fig. 6). Since Marchetti et al.^8^ reported that it was preferentially the expression of ACKR1 on the apical cell surface and not at junctions which correlated with transcellular migration, we quantified the staining intensity for ACKR1 at junctions and on the endothelial surface of cremaster and omentum venules. We found that ACKR1 expression was enriched at junctions of cremaster venules. Interestingly, junctional ACKR1 was similar in venules of the omentum and cremaster, whereas staining for ACKR1 on the endothelial surface was increased three-fold in venules of the omentum compared to venules of the cremaster (Fig. 6b).

We conclude that two cell surface proteins that have been associated with the transcellular diapedesis process are both expressed at higher levels on venules of the omentum. Although this alone is obviously not sufficient to tip the balance towards more transcellular diapedesis in WT mice, it may make venules in the omentum more prone to support transcellular migration in a situation where peripheral actin is more efficiently linked to junctional VE-cadherin.

### Stabilization of endothelial junctions interferes with endothelial cell elongation in the microvasculature of the omentum but not in cremaster

**Figure 7:**
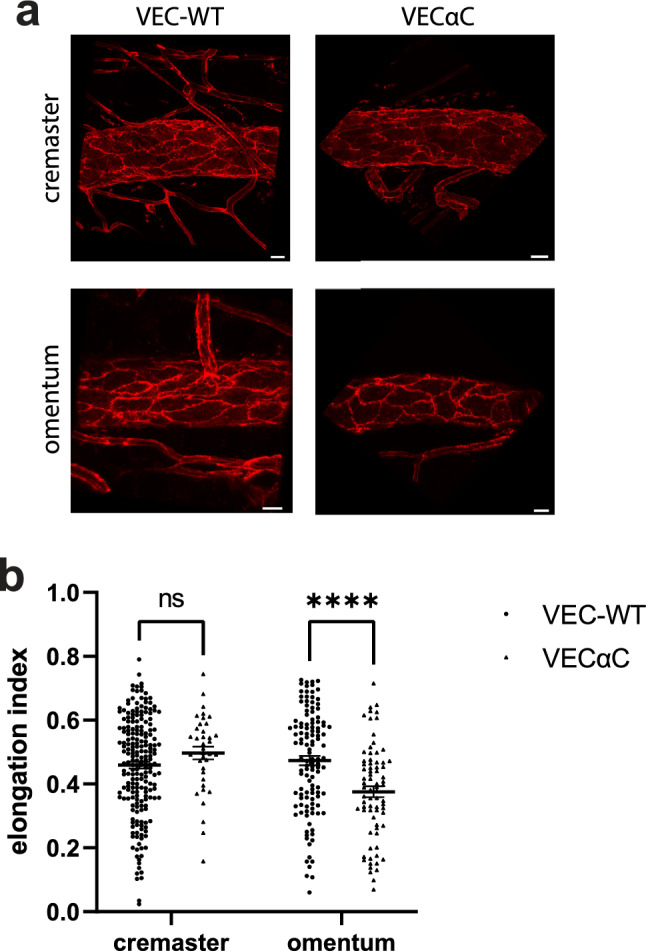
Endothelial cells are less elongated in venules of the omentum of VEC-αC mice. (**a**) Images of venule segments stained for PECAM-1 from cremaster muscle and omentum from VEC-WT and VEC-αC mice, bar = 20 µm. (**b**) Quantification of the elongation index of endothelial cells in venules (diameter 60–85 µm) of the cremaster and omentum of VEC-WT and VEC-αC mice. A total of 56 vessels (n = 20 VEC-WT cremaster, n = 17 VEC-αC cremaster, n = 11 VEC-WT omentum, n = 8 VEC-αC omentum) were evaluated and presented as mean ± SEM. ****P < 0.0001; two-way ANOVA.

To learn more about why VEC-αC drives neutrophils to migrate more avidly through the transcellular route of endothelial cells in the omentum, but not in the cremaster, we searched for differences between the venular endothelial cells in both tissues which would depend on the expression of VEC-αC. Remarkably, we noticed a difference in the morphology of endothelial cells between venules of the omentum and the cremaster muscle, which were only detected in VEC-αC mice, but not in WT mice (Fig. 7a). Only in omental venules of VEC-αC mice, EC were less elongated and more roundish, whereas endothelial cells in venules of the cremaster of VEC-αC mice and in venules of cremaster and omentum of WT mice were in all three cases more elongated and undistinguishable from each other, as determined by measuring the elongation index using ImageJ (Fig. 7b). Measurements were based on the analysis of at least 12 different vessel segments for each group.

Elongation of endothelial cells in the vasculature is influenced by mechanical forces based on the flow conditions within vessels. To investigate the reason why VEC-αC caused a reduction of endothelial cell elongation selectively in the omentum but not in the cremaster, we determined the blood flow in venules of both tissues and both genotypes. As shown in supplemental Fig. S1, we found no significant differences in blood flow velocity between the different groups. These results suggest that the expression of VEC-αC in combination with the mechanical properties of the omentum may modulate the biomechanical properties of endothelial cells in such a way that the elongation response to flow is impaired.

## Discussion

The VE-cadherin-α-catenin (VEC-αC) fusion protein binds more avidly to actin^18^ and stabilizes endothelial junctions. This leads to the inhibition of paracellular diapedesis of neutrophils through endothelial cells in vitro, blocks the inflammatory induction of vascular permeability in the skin and strongly inhibits neutrophil and lymphocyte extravasation in inflamed skin, lung and postcapillary venules of cremaster in VEC-αC knock in mice^14^. This suggests that extravasation in these organs occurs mainly through the paracellular diapedesis route and not transcellularly.

Remarkably, however, neutrophil recruitment into the inflamed peritoneal cavity is normal in VEC-αC mice. This prompted us to quantify neutrophil diapedesis through the transcellular and the paracellular pathway by intravital microscopy in the cremaster muscle and the omentum, the major site of neutrophil recruitment into the peritoneal cavity. In this way, we found: First, the paracellular diapedesis pathway is clearly the dominant route of neutrophil extravasation in the omentum as well as in the cremaster. Second, only in the omentum, VEC-αC causes an increase of transcellular migration, but not in the cremaster. Third, and most unexpected, in larger venules (60–85 µm diameter), VEC-αC does not inhibit paracellular diapedesis of neutrophils, neither in the omentum nor in the cremaster. This reveals, that the constitutive anchorage of VE-cadherin to junctional actin, affects the endothelial barrier function in various ways which depend on tissue specific qualities of the venules and even on venule caliber.

We found that about 80% of neutrophil accumulation in the peritoneal cavity within 3 h of IL-1β stimulation occurs through HEV-like venules in milky spots of the omentum, in line with earlier studies^20,21^. Since we also found that neutrophils extravasated from these vessels as efficiently in VEC-αC as in WT mice, it is likely that this is the main reason why VEC-αC fails to block extravasation into the peritoneal cavity. It is interesting, that lymph nodes were the only other tissue in VEC-αC mice, where leukocyte extravasation was unimpaired^14^. Like in the omentum, HEVs in lymph nodes are the main sites of leukocyte extravasation (lymphocyte homing). Thus, endothelial junctions of HEVs seem to be differently regulated in the context of leukocyte diapedesis.

The mechanism of transcellular diapedesis of leukocytes is not yet well understood. High expression levels of the atypical chemokine receptor 1 (ACKR1) and of ICAM-1 were demonstrated in vitro to correlate with an increase in transcellular transmigration^6,8,9^. In this context it is interesting that we found on endothelium of the omentum higher expression levels of these two cell surface receptors than in the cremaster muscle. Although this alone did not lead to more transcellular diapedesis in the omentum of WT mice, it may be a part of the reason why the expression of VEC-αC supports high levels of transcellular migration in venules of the omentum, but not in venules of the cremaster.

VEC-αC alone is not sufficient to increase the capability of endothelial cells to support transcellular migration. This follows from our results obtained in this study with the cremaster and from in vitro studies with HUVEC cells transfected with VEC-αC^14^. Likewise, the specific character of venular endothelium of the omentum of WT mice was also not sufficient to support transcellular diapedesis more avidly than endothelium in the cremaster. Therefore, we searched for differences between venular endothelium of omentum and cremaster which would depend on the expression of VEC-αC. The only such difference which we found was the reduced elongation of endothelial cells in omental venules, which was dependent on the expression of VEC-αC. Blood flow-induced shear stress is well known to drive elongation of endothelial cells. Since blood flow velocity was similar in omental and cremaster venules, and was independent of the expression of VEC-αC, we assume that VEC-αC may influence the responsiveness of endothelial cells to flow in an omentum specific way. Due to the fact that omentum is rich in adipocytes, it is possible that the mechanic properties of the omentum may vary from the cremaster muscle. It is conceivable that in such an environment the expression of VEC-αC which links VE-cadherin more strongly to the actin cytoskeleton^18^, may affect biomechanical properties of omental endothelium in such a way that the elongation response is impaired. At present, we do not yet know whether or how these effects are linked to the increased capability of VEC-αC expressing omental endothelial cells to support transcellular migration of neutrophils. However, changes in the biomechanical properties of endothelial cells were shown to affect the diapedesis process and the site of trafficking in various in vitro studies^22–25^. Thus, the changes in endothelial cell shape caused by VEC-αC expression specifically only in omental venular endothelium, may be taken as an indication for biomechanical properties that are specific for this endothelium and may affect the efficiency of transcellular diapedesis.

Given that VEC-αC clearly inhibits paracellular diapedesis in cultured endothelial cells and in vivo in postcapillary venules of the cremaster and in the skin and lung^14^, it was an unexpected finding that no inhibitory effect of VEC-αC could be detected for paracellular diapedesis of neutrophils in venules of larger caliber in the cremaster muscle and omentum. This argues for differences between postcapillary venules and larger venules with respect to the molecular mechanisms that control endothelial junction integrity. Differences in the responsiveness to barrier regulating mechanisms were also described between postcapillary venules and larger venules in the vasculature of the trachea^26^. In an allergy-based inflammation model it was found that vascular permeability for plasma components was induced at endothelial junctions of postcapillary venules whereas leukocyte extravasation occurred in larger venules^26^. Thus, either the molecular composition of adhesion mechanisms or the expression of adhesion regulating factors may vary between endothelial junctions of venules of different caliber.

Collectively, our direct comparison and quantification of paracellular and transcellular diapedesis in the omentum has revealed that paracellular diapedesis represents also in this tissue the dominant extravasation pathway for neutrophils. Yet, obstructing the opening of endothelial junctions with the VEC-αC fusion construct fails to impair neutrophil recruitment into the inflamed peritoneal cavity. This is mainly due to a failure of VEC-αC to block leukocyte extravasation through HEVs and to a smaller extent due to enhanced transcellular diapedesis in VEC-αC expressing larger venules of the omentum. Thus, unexpectedly, our results demonstrate that, depending on the type of venule, VEC-αC fails to inhibit paracellular diapedesis and can even enhance the efficiency of transcellular migration in venules of the omentum. Additional cell biological studies will be needed in the future to clarify, how the linkage of VE-cadherin to actin affects junction integrity and the transcellular passage of neutrophils.

## Materials and methods

### Mice

VEC-WT and VEC-αC mice, generated as previously described^14^ and LysM-eGFP mice^27^ were bred under pathogen-free conditions in the animal facility of the Max Planck Institute for Molecular Biomedicine. Mice were used at 11–20 weeks of age. General anesthesia was administered using Ketamine (125 mg/kg body weight) and Xylazine (12.5 mg/kg body weight) and mice were closely monitored throughout anesthesia. All experiments were carried out as approved by the local state authorities (Office for the Protection of Nature and Environment of the State of North-Rhine-Westphalia, Germany = Landesamt für Natur, Umwelt und Verbraucherschutz Nordrhein-Westfalen (LANUV)). Authors complied with the ARRIVE guidelines and all methods were performed in accordance with the relevant guidelines and regulations of LANUV.

### Antibodies

The antibody used for confocal intravital microscopy was anti-CD31 coupled to Alexa Fluor 555 (eBioscience, clone 390) and MECA79 against peripheral node addressin^28^ coupled to Alexa Fluor 647. The Antibodies used for whole mount staining were YNI-1.1 against ICAM-1 conjugated with Alexa Fluor 647^29^, anti-DARC against ACKR1 conjugated with Alexa Fluor 647^30^ (a gift from Ulrich von Andrian, Harvard Medical School, USA), and anti-PECAM-1 (5D2.6 and 1G5.1, in house)^31^.

### Neutrophil recruitment in peritoneal cavity

Peritonitis was induced with 50 ng IL-1β by intraperitoneal injections in VEC-WT or VEC-αC mice. In some experiments, the mAb MECA79 at an adhesion blocking dose (50 µg in 100 µl PBS) or a control antibody (IgM, abeomics, ABE-32-1368-10) were intravenously injected at the same time. 3 h later, mice were sacrificed and a peritoneal lavage was carried out using 20 ml PBS containing 3 mM EDTA. The peritoneal exudates were stained for CD45 (leukocytes, positive control), Gr-1^high^ (neutrophils) and IgG2b (negative control) and total numbers of infiltrating neutrophils per peritoneal cavity were determined by flow cytometry.

### Confocal intravital video microscopy

Intravital time-lapse videos were recorded via Zeiss LSM 880 with Airyscan Fast (20× Plan-Apochromat). VEC-WT and VEC-αC mice (12–14 weeks old) were irradiated and subsequently intravenously injected with bone marrow cells from LysM-eGFP mice. Six weeks later, mice were either intrascrotally or intraperitoneally stimulated with 50 ng IL-1β. Three hours later, endothelial cells were labeled by i.v. injection of anti-CD31 antibodies (30 µg/mouse) and in some experiments, HEVs were stained by i.v. injection of anti-MECA79 antibodies at a non-blocking dose (3 µg/mouse). For cremaster analysis, three hours later, mice were anaesthetized, placed in a heating chamber (35 °C) and the cremaster muscle was exteriorized and surgically prepared as previously described^16^. For analysis of the omentum, the tissue was exteriorized by a 1–1.5 cm median incision at the left upper abdomen and subsequently mounted onto a custom-designed microscopy stage with which the mouse was then transferred to a 35 °C heated chamber surrounding the LSM 880. For each mouse, Z-stack time lapse videos of 3 unbranched postcapillary venules with a diameter of 60–85 µm were recorded over a period of at least 20 min. Acquisition time per Z-stack did not exceed 30 s. For recording of extravasation through HEVs, video recordings started 2 h after IL-1β stimulation. Several vessels were recorded in one field of view (10× Plan-Apochromat). Z-stacks were acquired with an acquisition time of 23 s. Neutrophil extravasation was examined using IMARIS (Oxford Instruments, Abingdon, UK). For venules, neutrophil numbers were counted manually and are given per 450 µm vessel segment and 1 h. For HEVs the volume occupied by neutrophils outside of the vessel was determined in a volume of 237.92 µm × 237.92 µm × 54.09 µm around the selected vessel using IMARIS at 3 h after stimulation.

### Whole mount staining

Anesthetized mice were perfused with 10 ml of 4% paraformaldehyde (PFA) in PBS, followed by preparation of the omentum or cremaster muscle and fixation in 4% PFA for 1 h at room temperature. Subsequently, the tissues were blocked with 4% BSA/0.3% Triton X-100 overnight and incubated with primary antibody overnight followed by washing with PBST (PBS + 0.01% Triton X-100) and incubation with secondary antibody overnight. After 3 times of washing, tissues were embedded in DAKO fluorescent mounting medium and Z-stack projections were acquired using a Zeiss LSM 880 confocal microscope and analyzed with IMARIS. For quantification of junctional ACKR1 staining, PECAM-1 staining was used to create a mask of endothelial junctions from which the junctional ACKR1 fluorescence signal was quantified via IMARIS.

### Blood flow velocity measurement

To determine blood flow velocity, the motion of RBCs was determined by line-scan measurements at an LSM 880. Hereby, repetitive scans of the laser along the central axis of a postcapillary venule were performed. The displacement between consecutive longitudinal scans along the line allows for the calculation of the blood cell velocity. To visualize RBCs, we isolated erythrocytes from C57BL/6 WT mice and incubated them with 4 μM cell tracker Deep Red at 37 °C for 40 min. We injected 5% erythrocytes intravenously (5 erythrocytes per 100 erythrocytes in the donor mouse) into VEC-WT and VEC-αC mice^32^. Afterwards, mice were anesthetized and prepared for confocal IVM as described above. Line scans were performed with an average scanned distance of  < 100 µm, a temporal resolution of 1200 ms per scan and a record length of 50 µm. An ImageJ Macro was created to evaluate the recorded scans and determine the blood flow velocity^33–35^.

### Determining endothelial cell elongation index

Images were acquired from IVM videos. Endothelial cell morphology was visualized by PECAM-1 staining, and cell shapes were fitted to ellipses using ImageJ (Analyze Particles). The elongation index was determined by the formula: major axis length − minor axis length divided by major axis length + minor axis length.

### Statistical analysis

Mice of both genotypes were randomly assigned to groups. Analysis was done in a blinded fashion. Data are presented as mean ± standard error of the mean (SEM). Normally distributed data was statistically analyzed by unpaired *t* test, one-way ANOVA or two-way ANOVA, using the GraphPad Prism Software. P-values below 0.05 (*), 0.01 (**), 0.001 (***) and 0.0001 (****) were considered statistically significant.

### Supplementary Information

Supplementary Figure S1.

Supplementary Video 1.

Supplementary Video 2.

Supplementary Video 3.

Supplementary Video 4.

Supplementary Video 5.

Supplementary Video 6.

Supplementary Video 7.

Supplementary Legends.

## Data Availability

The datasets used and/or analyzed in the current study are available from the corresponding author upon reasonable request.
