# Supplementary material for: Conditions that promote transcellular neutrophil migration in vivo
Source: Sci Rep. 2024 Jun 24;14:14471. doi: 10.1038/s41598-024-65173-3 (PMC11196655; doi:10.1038/s41598-024-65173-3)
Supplement: Supplementary file 1 — Supplementary Figure S1. [file 41598_2024_65173_MOESM1_ESM.pdf]

Conditions that promote transcellular neutrophil migration in vivo

Min Xia, Rebekka I. Stegmeyer, Keisuke Shirakura, Stefan Butz, Aude Thiriot, Ulrich H. von Andrian and Dietmar Vestweber

Supplemental Fig. S1

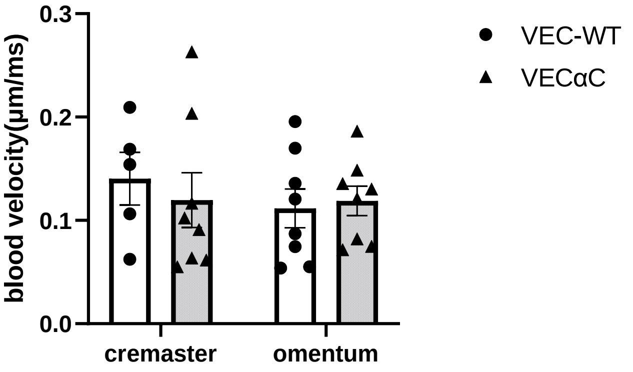

Supplemental Fig. S1: Blood velocity measurement.

Blood flow velocity was determined in venules (diameter 60-85 µm) of the cremaster and omentum of VEC-WT and VEC-alphaC mice as described in Materials & Methods.

Data were determined from at least 5 mice per group and are presented as mean ± SEM.
