# Supplementary material for: Conditions that promote transcellular neutrophil migration in vivo
Source: Sci Rep. 2024 Jun 24;14:14471. doi: 10.1038/s41598-024-65173-3 (PMC11196655; doi:10.1038/s41598-024-65173-3)
Supplement: Supplementary file 9 — Supplementary Legends. [file 41598_2024_65173_MOESM9_ESM.docx]

**Video legends**

**Suppl. video 1:**

Neutrophil extravasation in the omentum of mice, 3 h after i.p. stimulation with 50 ng IL-1β. All vessels are stained for PECAM-1 (red), high endothelial venules (HEV) in milky spots by MECA-79 (magenta) and neutrophils genetically labeled by LysM-EGFP (green). Note that neutrophil extravasation is initiated in HEVs first and followed later by the extravasation of neutrophils in other venules.

**Suppl. video 2:**

Overview of numerous extravasation events (3h after i.p. injection of 50 ng IL-1β) in a venule segment of the omentum of WT mice, stained for PECAM-1 (red) neutrophils (LysM-eGFP). Arrowheads point to paracellular extravasation events. Bar=15 μm. Frame rate =24 fps. Imaging duration is 20 minutes and the elapsed time between two images is 30 seconds.

**Suppl. video 3:**

Same video as suppl. video 2 omitting the green signal.

**Suppl. video 4:**

Zoom-in taken from video 2/3 to more closely visualize a paracellular transmigration event (arrowhead) in a short vessel segment of an omental vessel stained with anti-CD31 (red) and neutrophils are visualized in green (LysM-eGFP). Bar=15 μm. Frame rate =24 fps. The video depicts an imaging duration of 8 minutes and the elapsed time between two images is 30 seconds.

**Suppl. video 5:**

Same video as suppl. video 4 omitting the green signal.

**Suppl. video 6:**

Zoom-in taken from a confocal IVM movie to more closely visualize a transcellular transmigration event (arrowhead) in a short vessel segment, 3 hours after IL-1β stimulation. EC junctions were stained with an anti-CD31 antibody (red) and neutrophils visualized in green (Ly-EGFP). Bar=20 μm. Frame rate =24 fps. The video depicts an imaging duration of 3 minutes and the elapsed time between two images is 24 seconds.

**Suppl. video 7:**

Same video as suppl. video 6 omitting the green signal.
